# Correction: Klauser et al. Line Tension and Drop Size Dependence of Contact Angle at the Nanoscale. *Nanomaterials* 2022, *12*, 369

**DOI:** 10.3390/nano13212847

**Published:** 2023-10-27

**Authors:** Waldemar Klauser, Fabian T. von Kleist-Retzow, Sergej Fatikow

**Affiliations:** Division Microrobotics and Control Engineering, Department of Computing Science, University of Oldenburg, D-26129 Oldenburg, Germany

## 1. Text Correction

There was an unclear passage in the original publication [1]. The value of the line tension is actually negative. This is clearly seen in Figure 3b. While it is implicit, we would like to point out that the value of the line tension we give is an absolute value, and we would like to add a minus to the value range extracted from Figure 3b.

A correction has been made to “*Section 3*, *Results*”:

“From this fit, we determined an absolute value for the apparent line tension, *τ* = 4.02 × 10^−7^ J/m. Furthermore, for better display of the validity of the measurement results, *θ_C_* versus one divided by the radius of the spheres was plotted too (see Figure 3b). The solid red line corresponds to the previously determined *τ*. The two additional dotted lines correspond to 2 *τ* and 1/2 *τ*. Thus, more than 90% of the data points are located in the funnel that spans between −8.04 × 10^−7^ J/m < *τ* < −2.01 × 10^−7^ J/m.”

## 2. Reference Correction

In the original publication, Ref. [22] was not cited correctly, it should change as follows:

New Ref. 22: Zhao, B.; Luo, S.; Bonaccurso, E.; Auernhammer, G.K.; Deng, X.; Li, Z.; Chen, L. Resolving the Apparent Line Tension of Sessile Droplets and Understanding its Sign Change at a Critical Wetting Angle. *Phys. Rev. Lett.* **2019**, *123*, 94501

The authors apologize for any inconvenience caused and state that the scientific conclusions are unaffected. This correction was approved by the Academic Editor. The original publication has also been updated.
