# Supplementary material for: Line Tension and Drop Size Dependence of Contact Angle at the Nanoscale
Source: Nanomaterials (Basel). 2022 Jan 24;12(3):369. doi: 10.3390/nano12030369 (PMC8840433; doi:10.3390/nano12030369)
Supplement: Supplementary file 1 [file nanomaterials-12-00369-s001.zip › nanomaterials-1540970-supplementary.pdf]

Supplementary Materials

Line Tension and Drop Size Dependence of Contact Angle at the Nanoscale

Waldemar Klauser *, Fabian T. von Kleist-Retzow and Sergej Fatikow

Division Microrobotics and Control Engineering, Department of Computing Science, University of Oldenburg, D-26129 Oldenburg, Germany; fabian.von.kleist-retzow@uni-oldenburg.de (F.T.v.K.-R.); sergej.fatikow@uni-oldenburg.de (S.F.)

* Correspondence: waldemar.klauser@uni-oldenburg.de

Figure S1 shows two liquid metal manipulators. Figure S1 a shows the error described if the tip was also in the ion beam during FIB milling of the sphere. As a result, the wetting of the tip continues and the droplet may adhere to the tip in an elongated manner. After this failure occurs, all liquid metal must be removed using FIB or a new tip must be fabricated. Only by completely removing the liquid metal can the wetting be reversed and the contact point renewed.

Figure S1 b shows a freshly manufactured manipulator with a sphere diameter of 44 µm. It can be clearly seen that there is still no oxide on the sphere. This configuration of tungsten tip and liquid metal sphere is ideal for contact angle measurements. Because the sphere is fixed to the side of the tip, it can be easily reduced in size by FIB milling.

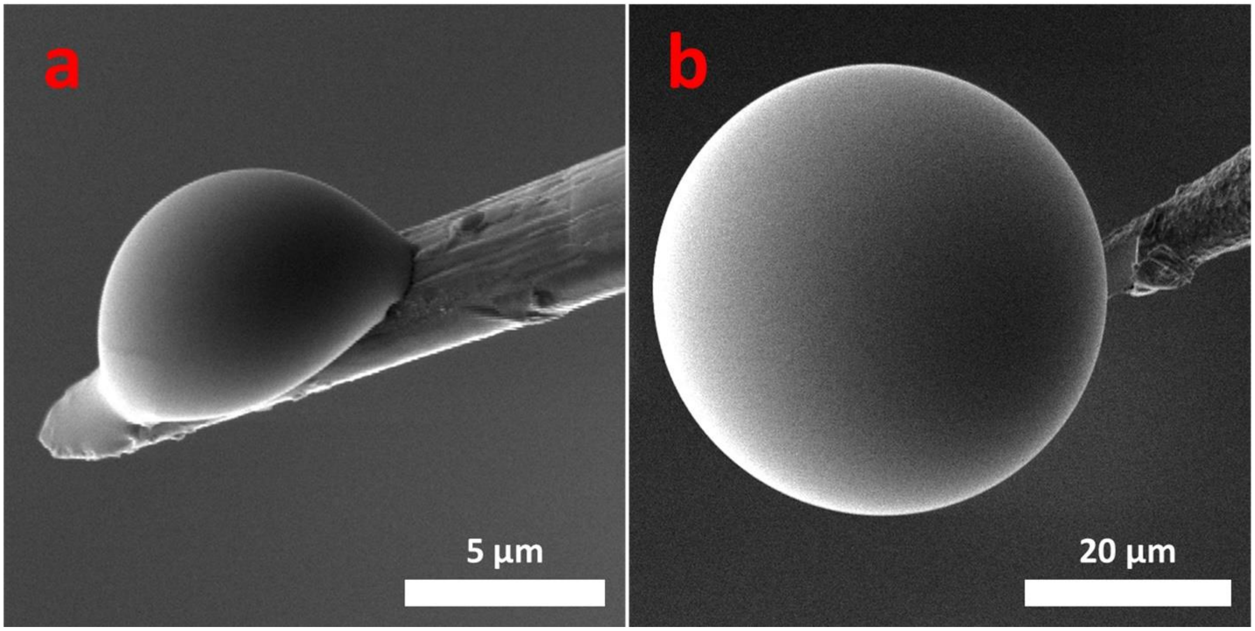

Figure S1. SEM micrographs of two liquid metal manipulators showing a) a defective tip through unwanted wetting and b) an ideal manipulator for the presented measurement series.
